# Sex and STEM Occupation Predict Autism-Spectrum Quotient (AQ) Scores in Half a Million People

**DOI:** 10.1371/journal.pone.0141229

**Published:** 2015-10-21

**Authors:** Emily Ruzich, Carrie Allison, Bhismadev Chakrabarti, Paula Smith, Henry Musto, Howard Ring, Simon Baron-Cohen

**Affiliations:** 1 Autism Research Centre, Department of Psychiatry, University of Cambridge, Cambridge, United Kingdom; 2 Cambridge Intellectual and Developmental Disabilities Research Group, Department of Psychiatry, University of Cambridge, Cambridge, United Kingdom; 3 NIHR CLAHRC-EoE for Cambridgeshire and Peterborough NHS Foundation Trust, Cambridge, United Kingdom; 4 Centre for Integrative Neuroscience and Neurodynamics, School of Psychology and Clinical Language Sciences, University of Reading, Reading, United Kingdom; 5 CLASS Clinic, Cambridgeshire and Peterborough NHS Foundation Trust, Cambridge, United Kingdom; Osaka University, JAPAN

## Abstract

This study assesses Autism-Spectrum Quotient (AQ) scores in a ‘big data’ sample collected through the UK Channel 4 television website, following the broadcasting of a medical education program. We examine correlations between the AQ and age, sex, occupation, and UK geographic region in 450,394 individuals. We predicted that age and geography would not be correlated with AQ, whilst sex and occupation would have a correlation. Mean AQ for the total sample score was m = 19.83 (SD = 8.71), slightly higher than a previous systematic review of 6,900 individuals in a non-clinical sample (mean of means = 16.94) This likely reflects that this big-data sample includes individuals with autism who in the systematic review score much higher (mean of means = 35.19). As predicted, sex and occupation differences were observed: on average, males (m = 21.55, SD = 8.82) scored higher than females (m = 18.95; SD = 8.52), and individuals working in a STEM career (m = 21.92, SD = 8.92) scored higher than individuals non-STEM careers (m = 18.92, SD = 8.48). Also as predicted, age and geographic region were not meaningfully correlated with AQ. These results support previous findings relating to sex and STEM careers in the largest set of individuals for which AQ scores have been reported and suggest the AQ is a useful self-report measure of autistic traits.

## Introduction

The Autism-Spectrum Quotient (AQ) is a brief self-report measure suitable for adults with average or above average IQ [1]. It has been used both in research [2] and to aid in clinical practice, during referral to specialist autism diagnostic clinics [3]. The AQ has also been used in the general population to quantify autistic traits along a quantitative dimension of individual differences [2]. Here we examine the distribution of AQ scores in a very large population sample, collected online, by Channel 4, a UK national television production company.

Before describing the rationale for the study, it is important to acknowledge the benefits [4] and the risks [5] of ‘big data’. The term ‘big data’ refers to large sets of digital records, and to a developing research paradigm [6,7]. The analysis of big data is now common in business and finance, social media, advertising, medicine and epidemiology (see for example Google flu trends [8] and the recent Ebola outbreak [9]). Big data can be particularly useful to examine the relationship of seemingly unrelated variables, such as in a recent study of adverse drug reactions via social media [10]. Big data also has appeared in pure research, such as genome sequencing or neuroimaging to uncover the human connectome. Big data has the potential to test the robustness of previous findings but one needs to be aware not just of volume (data size) and velocity (rate of data accrual) [11], but also variety (data type and scope), value (data worth), and veracity (the reliability of the data) [7,12]. Drawbacks of big data include ethical considerations of privacy and security, as well as storage and processing limitations [7,13,14]. In addition, standard statistical methods may not be appropriate for use with big data [7], and the ability of big data studies to identify subtle correlations often considered a strength, can sometimes be disadvantageous [15]. There is a tendency to view big data as accurate and objective, without considering potential biases in data collection and cleaning [5]. Large sample sizes have the potential to amplify error, so care needs to be taken in data interpretation to clarify in what way large samples may not be representative. Many of these drawbacks can be overcome as needed, for instance by planning for heavy computational processing, using specialised storage and visualisation platforms, using scaling algorithms or other adjustments, correcting for multiple comparisons, calculating effect size in addition to significance, and thoroughly characterising the population sample to ensure sample representativeness.

With these caveats in mind, the present study uses big data to examine correlations between the AQ and selected available demographic variables (age, sex, occupation, and UK geographic region). We examined a very large set of AQ data collected following the screening of the UK Channel 4 television program *Embarrassing Bodies*: *Live from the Clinic*. A study of this nature is important because it offers the opportunity to test to findings reported in smaller samples. Secondly, it offers the opportunity to investigate the AQ in a large data set, since signals of small effect size might not be apparent in smaller samples. Finally, it offers the opportunity to use regression modelling to disentangle the influence of several factors on AQ. We hypothesized that sex and occupation would predict autistic traits, whilst age and geographical region would not. These predictions were based on previous research showing that, in the general population, males on average have higher AQ scores than do females [16]. In addition, individuals who work in fields that require high ‘systemizing’ (the drive to analyse or build rule-based systems) such as science, technology, engineering, and mathematics (STEM), show higher levels of autistic traits as measured by AQ than do those working in non-STEM fields [1,17–20]. The increased prevalence of individuals with autism in regions that are rich in jobs in Information Technology (IT) [21] also leads to the prediction that AQ will be higher in those working in STEM than in non-STEM jobs. In contrast, previous studies have not found that AQ is influenced by age and whilst small fluctuations in AQ have been reported across cultures [22,23], these may reflect subtle differences in the meanings in translations of items into languages (the AQ has now been translated into approximately 30 languages (http://www.autismresearchcentre.com/arc_tests), and we had no reason to expect that geographic region within the UK would correlate with AQ.

## Materials and Methods

Details of the development of the Autism-Spectrum Quotient (AQ) can be found elsewhere [1]. The AQ is a 50-item self-report measure for characterizing an individual’s degree of autistic traits [1]. The AQ includes questions evaluating both social and non-social domains and examines behaviour, cognition, ability, and preference in a brief, self-administered, forced-choice format. Individuals are instructed to respond to each of the 50 items with one of four responses: ‘definitely agree’, ‘slightly agree’, ‘slightly disagree’, and ‘definitely disagree’. Questions are counterbalanced to avoid a response bias, so that half of the ‘agree’ responses and half of the ‘disagree’ responses are scored as an autistic trait. These responses are scored using a binary system, where an endorsement of the autistic-like behaviour (either mildly or strongly) is scored as a +1, while the opposite response is scored as a 0, leading to a maximum score on the AQ of 50. Using this scoring system, it is evident that autistic traits exist along a continuum that extends from the typical population to clinically diagnosed individuals with autism spectrum conditions (ASC).

AQ scores are normally distributed and the AQ demonstrates good test-retest reliability and internal consistency [1]. The AQ has acceptably high sensitivity and specificity: at a cut-off score of 26, 83% of people referred to an adult autism clinic were correctly identified (sensitivity 0.95, specificity 0.52, positive predictive value 0.84, negative predictive value 0.78), while a cut-off score of 32 was found to correctly identify 76% of people (sensitivity 0.77, specificity 0.74) [3]. The AQ was designed for adults with average IQ or above [1], so is suitable for use in the general population and for at least 50% of people on the autism spectrum [24]. The questionnaire is not suitable for individuals with low IQ or language impairment, as it relies on the comprehension of the 50 questions.

### Data collection

The Channel 4 TV program *Embarrassing Bodies*: *Live from the Clinic* has a website with a number of self-report measures related to psychological and psychiatric traits (http://mindchecker.channel4.com/). This online self-assessment was launched during season 4 of the program. In March, the AQ was uploaded as the ‘Autism Test’ on this site, and on 22 April 2014 the AQ was officially launched for the first broadcast of an episode that included a brief TV segment following an individual with Asperger Syndrome [25]. In the episode, the TV presenters (both medical doctors) introduced the topic of autism by giving population prevalence estimates, and explained that many individuals in the general population have autistic traits. They invited viewers by saying, “Why not go onto our website and take the My MindChecker test to measure the extent of any autistic traits you might have, and whilst your results are confidential, the overall data will form the biggest national survey of its kind.” The program continued by explaining the symptoms of autism: difficulty with social interaction, trouble understanding aspects of humour such as sarcasm, feelings of anxiety associated with sensory stimulation, and intense special interests and hobbies. The presenters concluded, “If you are experiencing similar symptoms […] and want to find out if you have a condition that affects how you interact with the world and other people, then take part now in our self test.” The link to the data collection site was displayed at the outset of the autism segment and at intervals throughout the episode. The presenters announced approximately 30 minutes after the initial mention of the website that 40,000 individuals had already completed the survey. Further, according to the site’s builder, in a blog entry from 22 April 2014, the AQ “was done 63,000 times during the hour of the show and by 11pm (3 hours in) that had reached 100,000. The total [eight days after the site launch] stands at 350,000 completed tests.” [26]. Since the initial call for participants, the site has undergone several updates and improvements, including the addition of a disclaimer—at the request of our research team—that the AQ is not a diagnostic test for autism. However, the AQ itself remained unchanged in its content during these changes in the website design. All data was anonymous.

### Data extraction

After the original broadcast, the research team contacted the show producer to request access to the anonymized data. Ethical approval for use of the anonymized data was obtained from the Psychology Research Ethics Committee (PREC), University of Cambridge, UK. Consent was obtained online when participants agreed to Channel 4’s terms and conditions before beginning the questionnaire. These terms and conditions included a description of how the anonymized data would be used for research, explicitly stating: “What will your data be used for? In future, anonymized test results may be available through a public API which will allow access to the anonymized data for analysis by approved third parties.” As the data were collected anonymously, the participants did not have any direct contact with the investigators, and no personally identifiable information at any stage was available to either the investigating team or the Channel 4 website hosting the questionnaire. Data were extracted and transferred to the research team on 5^th^ June 2014, effectively restricting the sample size to this date. Data transferred included an anonymized ID number for each questionnaire taken, the date each entry was created (ranging from 28^th^ March 2014 to 5^th^ June 2014), AQ total score (using the standard scoring method out of 50), and the respondent’s sex, age, occupation, and UK region of residence, selected by the participant via dropdown menus. In addition, participants were asked to respond to a question about perceived autism prevalence. Importantly, participants were not asked about whether they, or a family member, had a diagnosis of ASC, a limitation that we return to discuss later. We have requested this item be added to the website, so that a future replication of the current study compare AQ scores of those with and without ASC. Even without this variable, the large dataset presents a rare opportunity to test our predictions.

### Participant characterization

The original dataset transferred by Channel 4 consisted of 514,972 entries. Entries were excluded if participants reported their age to be under 16 or over 89 years old. These age limits were used because the AQ is designed as a self-report instrument for adults; 16 years old was selected based on the lower age limit of the original AQ sample [1]. The upper range from the Channel 4 dataset was 120 years old; 89 was selected as an upper age limit to correspond with other recommended upper age limits in other cognitive tests, such as the Wechsler Abbreviated Scale of Intelligence (WASI) [27]. In addition, individuals who selected the response “Prefer not to say” in response to the questions about sex, occupation, and region of residence were removed, to assist later data interpretation. There was no meaningful difference in mean AQ scores between the data that were excluded and the retained data. The final sample was n = 450,394. To contextualize the data [5] we characterized the distributions of each of the demographic variables in relation to the general population prior to any analysis of AQ scores, using R [28].

#### Age and sex

Number of individuals stratified by sex and mean age are shown in Table 1.

**Table 1:** Participant characteristics.

	N (%)	Mean age (SD)
Female	298,084 (66.2)	36.69 (13.61)
Male	152,310 (33.8)	37.73 (14.10)

**Fig 1:**
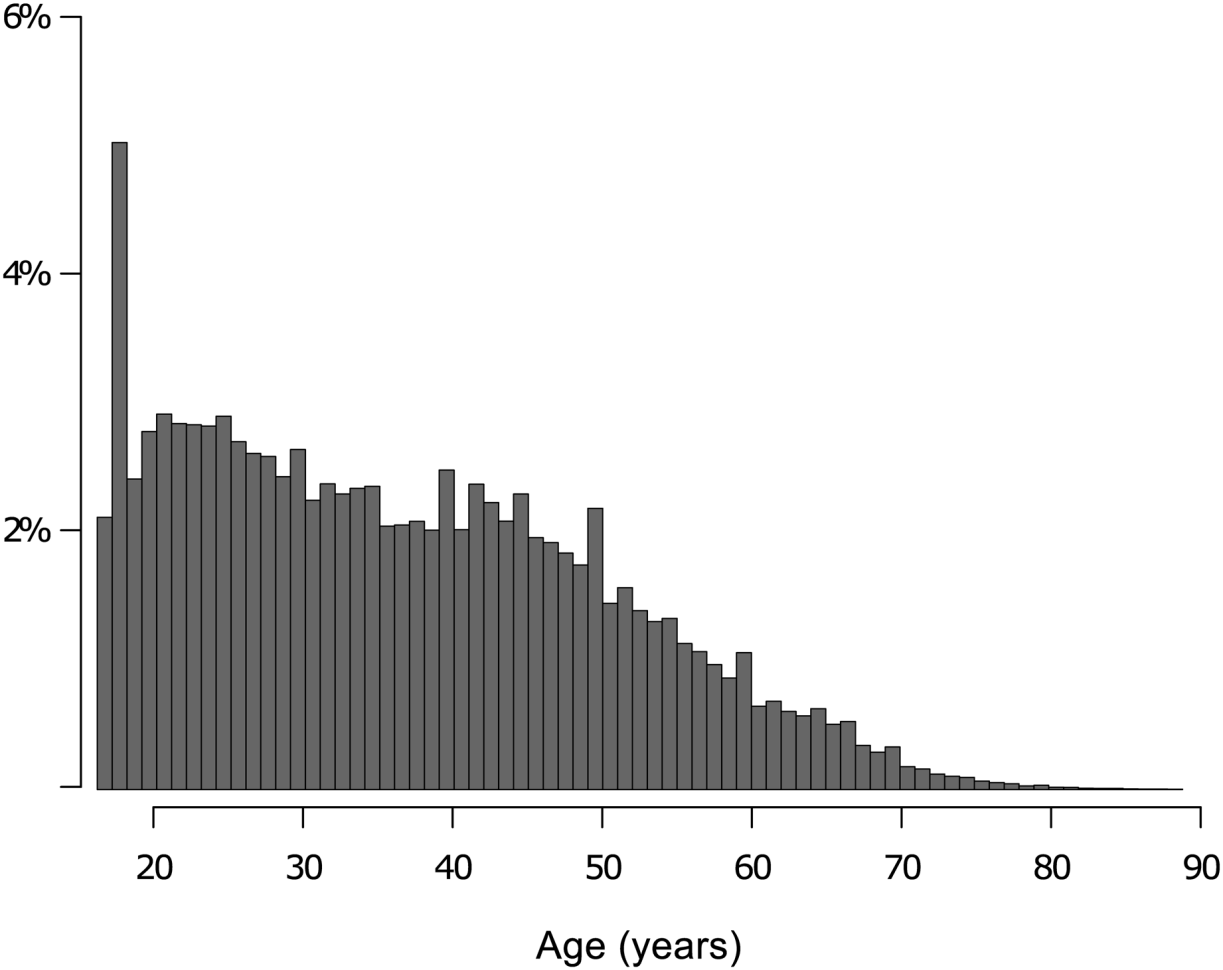
Age distribution of sample. Histogram of the relative frequency of surveys taken by reported age of participant.

As can be seen in Fig 1, age is skewed to a younger population (skew = 0.50, kurtosis = -0.32), as might be expected in relation to the demographic of viewers of a late-night Channel 4 TV program. However, there are substantial numbers of respondents reporting ages of up to 50 years. Direct comparisons of the current sample to viewer demographics were not conducted because data were not available from the broadcasting company.

A Welch’s 2-sample t-test was conducted to compare mean age in males and females. Although there was a statistically significant difference in age between males and females: t(297,392) = 23.61, *p* < 0.001, 95% CI [0.95, 1.12], the effect size was extremely small, *d* = 0.08, implying the difference is not meaningful, and only reaches statistical significance due to the large sample size.

#### Region of residence

Six selection options were: (geographic region) Northern England, Northern Ireland, Scotland, Southern England, Wales, and Other. The Office for National Statistics (http://www.ons.gov.uk/) was queried for regional population estimates. The regions from the Population Estimates for UK, England and Wales, Scotland and Northern Ireland, Mid-2013 were equated with the regions from the dataset (see Table 2).

**Table 2:** Data region categories from the Office for National Statistics and the Channel 4 program. Note that “Other” may not exclusively apply to the ONS categories, described as individuals living in the Midlands and East of England.

Channel 4 categories	Mapped ONS categories
Northern England	North East, North West, Yorkshire and The Humber
Southern England	London, South East, South West
Wales	Wales
Northern Ireland	Northern Ireland
Scotland	Scotland
Other	East Midlands, West Midlands, East of England

**Fig 2:**
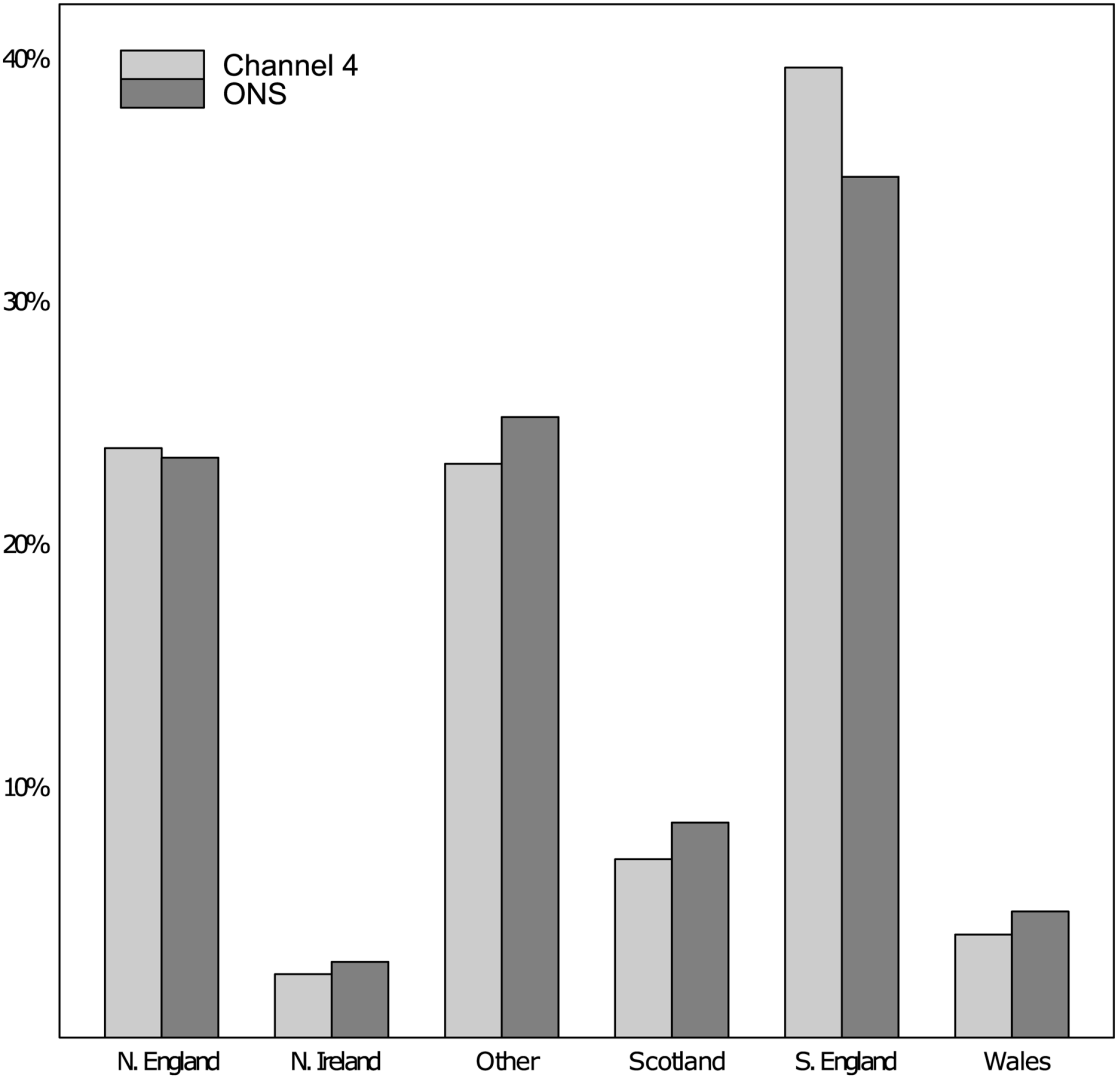
Bar plot of reported regions of residence taken from National Statistics Board as compared to current survey.

Pearson’s Chi-squared tests were performed. The proportion of individuals that were from a particular UK region as reported by the ONS did not significantly differ from the proportions that responded to the Channel 4 survey (Fig 2). This suggests that a representative population sample was obtained in terms of numbers from the distributed regions of the UK.

#### Occupation

21 selection options were: (occupation) Civil Engineering, Computers & I.T., Director, Engineering, Financial Banking, Food & Drinks, Healthcare, Hospitality, Legal, Leisure, Office Administration, Public Sector Services, Publishing & Media, Retail, Sales, Scientific & Technical, Supply Chain, Teaching, Translation & Interpretation, Transport, and Other. STEM occupations (Science, Technology, Engineering, and Mathematics) were differentiated from non-STEM occupations (see Table 3).

**Table 3:** List of occupations.

STEM occupations	Non-STEM occupations
Computers & I.T.	Civil Engineering
Engineering	Director
Scientific & Technical	Financial Banking
	Food & Drinks
	Healthcare
	Hospitality
	Legal
	Leisure
	Office Administration
	Public Sector Services
	Publishing & Media
	Retail
	Sales
	Supply Chain
	Teaching
	Translation & Interpretation
	Transport

**Fig 3:**
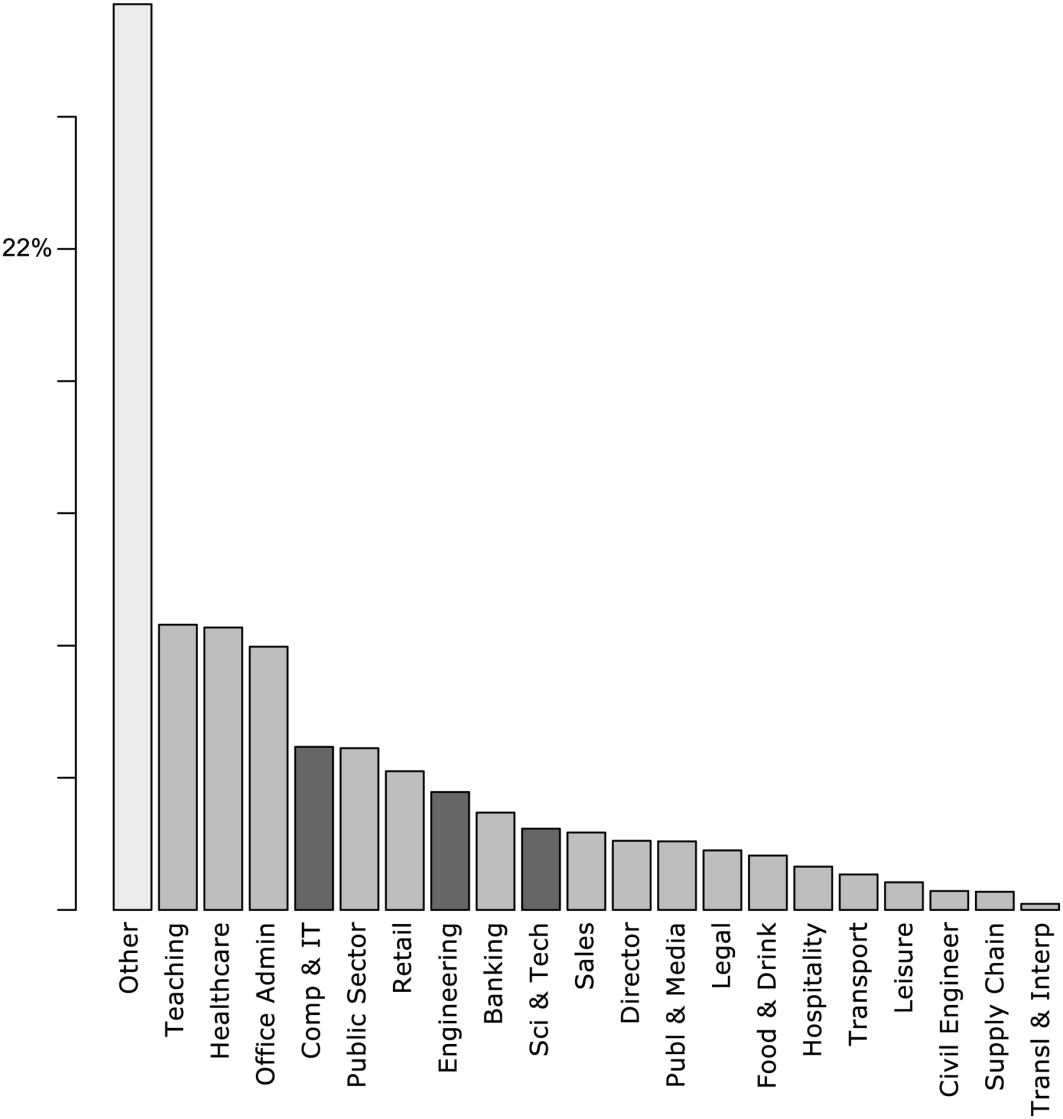
Bar plot of reported occupations sorted by relative frequency. STEM careers depicted in dark grey, non-STEM depicted in grey, Other depicted in light grey.

The relative frequencies of reported occupations can be found in Fig 3.

### Data analysis

Once the sample was cleaned and characterized, statistical significance and effect size (practical significance) were calculated for the relationship between AQ and each demographic variable, independent of the effect of the other variables. Regression analyses were then performed to investigate the respective contributions of each of the measured demographic variables to the overall variance, to identify interaction terms, and to examine the standard AQ cut-off point reported in the literature. Stepwise model selection analyses were conducted to establish which combination of the available variables best explains the variance in AQ scores. Binary logistic regression was conducted to examine the extent to which demographic variables predicted a high-risk group as defined by AQ score. Analysis of AQ was also performed using R [28].

## Results

### Raw AQ distribution

**Fig 4:**
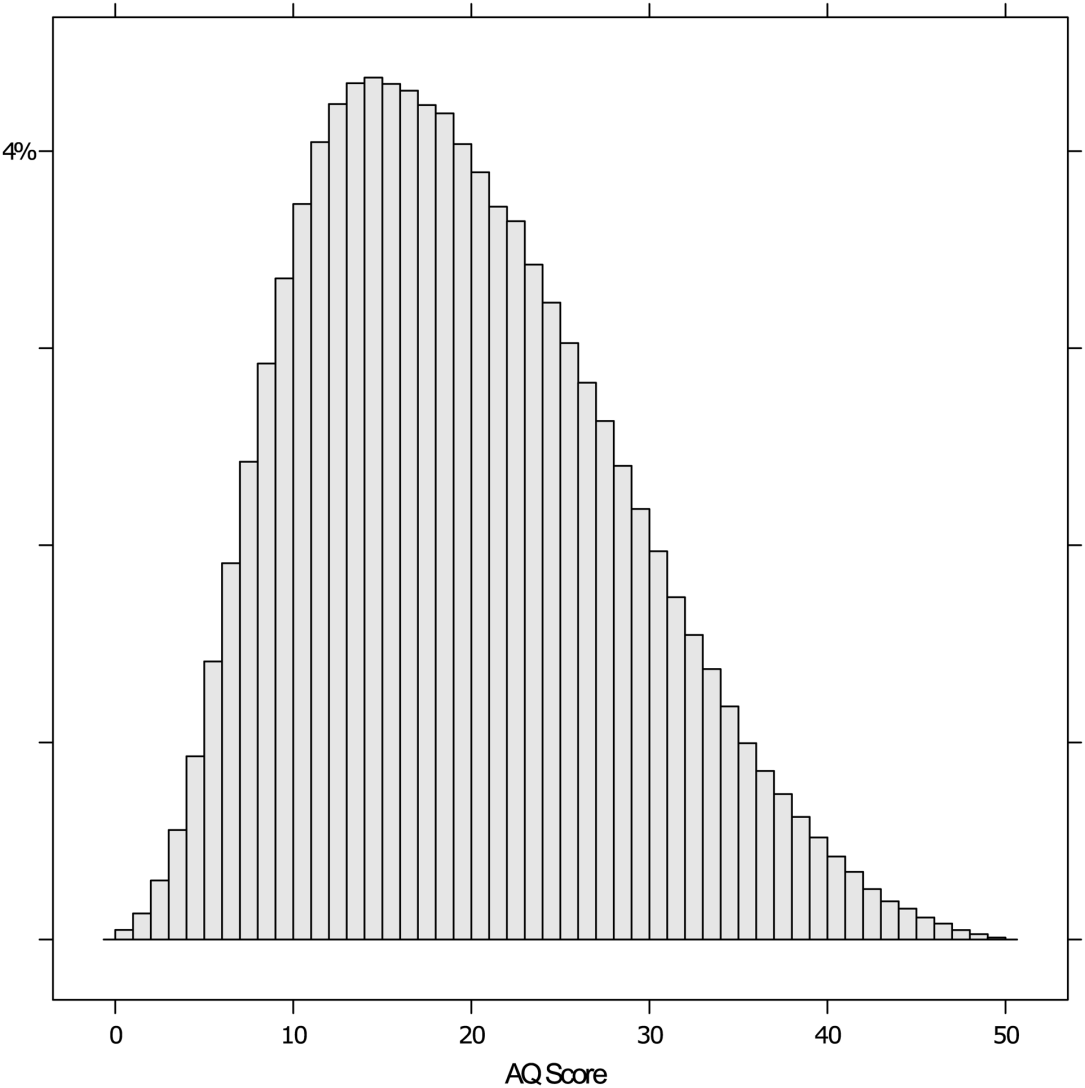
Histogram of the AQ scores.

From the data (n = 450,394), the reported mean AQ score was 19.83 (SD = 8.71), and tests of normality (Kolmogorov-Smirnov and Anderson-Darling) revealed this data had a positive skew of 0.47 and a kurtosis of -0.27 (See Fig 4).

AQ scores for each demographic variable were calculated (Table 4). All differences were statistically significant. However, in an effort to distinguish statistical significance from practical relevance, effect sizes were also calculated. For t-tests and correlations, Cohen’s *d* was calculated; for ANOVAS, eta squared was calculated and then converted to *d* as described by [29]. By convention, *d* = 0.2 is a 'small' effect size, 0.5 is a 'medium' effect size and 0.8 is a 'large' effect size. For Sex, *d* = 0.3. For age (continuous variable), *d* = 0.1. Cohen’s *d* was negligible for Location (*d* = 0.06), making it a poor predictor of AQ scores, as is clear from the minimal difference in mean regional AQ scores. For Occupation, the effect size when STEM and non-STEM careers are compared was *d* = 0.26; this value was not appreciably reduced when Other was included along with STEM and non-STEM categories.

**Table 4:** AQ scores by demographic group. Age, treated as a continuous variable, has been stratified for ease of presentation.

	Group	AQ Mean (SD)	CI
**Sex**	Male	21.55 (8.82)	21.50–21.59
	Female	18.95 (8.52)	18.92–18.98
**Age**	Young adults (16–35)	20.08 (8.56)	20.04–20.11
	Middle aged (36–64)	19.63 (8.88)	19.59–19.66
	Elderly (65+)	18.85 (8.51)	18.72–18.98
**Region**	S. England	19.61 (8.78)	19.57–19.65
	N. England	20.05 (8.72)	20.00–20.10
	Wales	20.42 (8.66)	20.30–20.55
	Scotland	19.62 (8.77)	19.52–19.72
	N. Ireland	19.66 (8.53)	19.49–19.82
	Other	19.94 (8.61)	19.97–20.07
**Occupation**	STEM	21.92 (8.92)	21.85–22.00
	non-STEM	18.92 (8.48)	18.88–18.95
	Other	20.68 (8.58)	19.89–20.00

**Fig 5:**
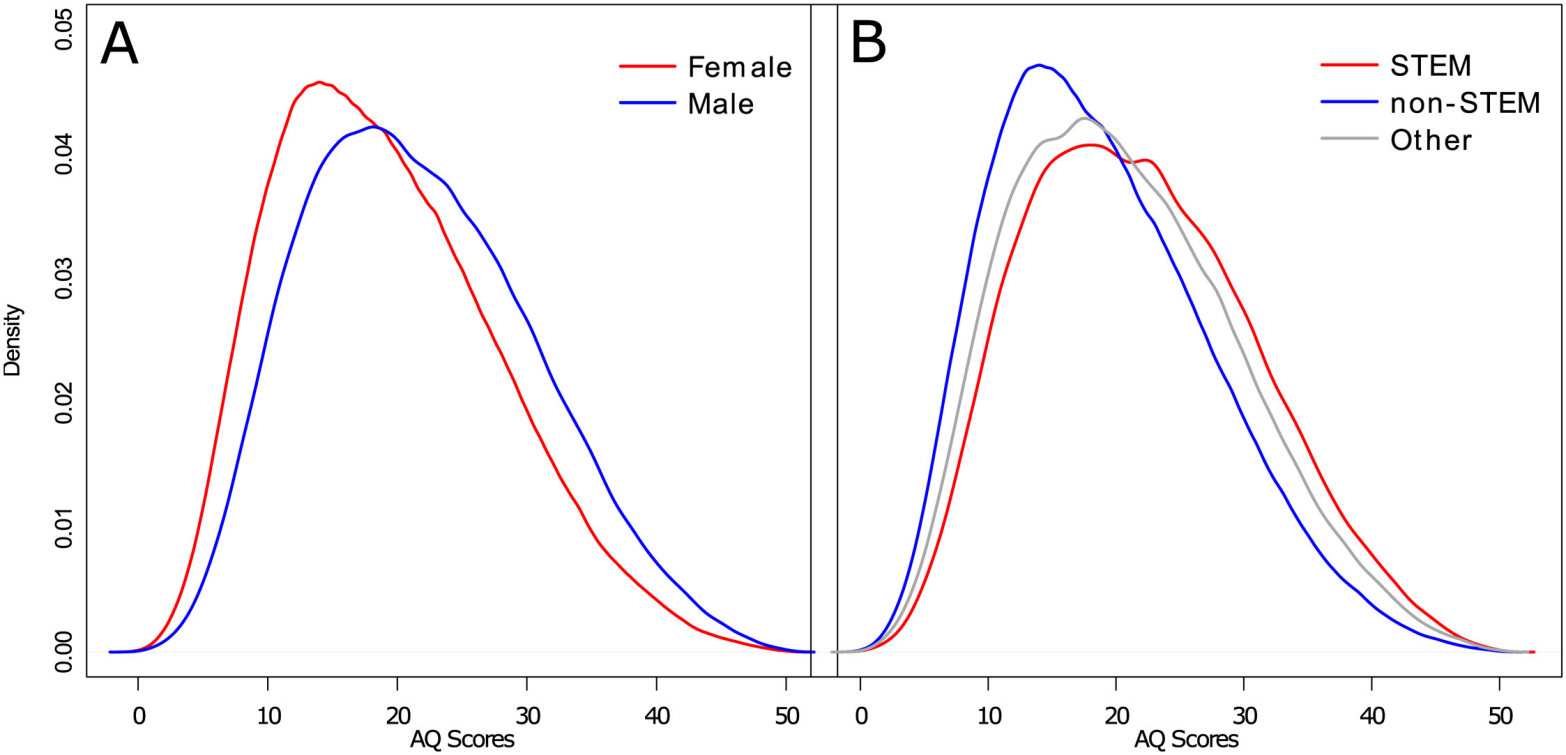
Density plots for selected hypothesis-driven demographic variables. A. Sex. B. Occupation.

Density plots of Sex and STEM on AQ scores are shown in Fig 5.

### Multiple linear regression

In addition to determining the effect of each of the demographic variables in isolation, stepwise multiple linear regression analysis was performed, to examine the combined extent to which the measured variables explained the variance within the sample. Terms included in analysis were Sex (Male vs. Female), Age (continuous), Occupation (STEM vs. non-STEM vs. Other). The Location term was excluded from this analysis for several reasons: the effect size of this variable in independent analysis was negligible, the ambiguous and coarse-grained manner in which the data were collected (e.g., “Other”; lack of Midlands and London regions), and the lack of literature support of a geographic effect on autistic traits. To determine multivariate outliers, Mahalanobis distance, or the distance between a distribution and an individual data point was calculated, and 6 individuals were removed from analysis due to a score of greater than 20.5 (all individuals were around the age of 88, 5 of 6 were males, all were STEM, and there was no trend for AQ score).

The fit of the regression model relating the covariates to AQ score is shown in Table 5. With R^2^ = 0.036 (adjusted R^2^ also 0.036; model generalizes very well due to large sample size), very little variance in AQ scores—only 3.6%—is explained by the covariates included in the model. Unsurprisingly, given the very large statistical power in this sample, most terms are significant; thus, it is important to consider effect sizes (semi-partial correlation r) when interpreting the results. Ignoring the effect of any covariates, the average AQ score is 20.35 (intercept value). There are significant interactions between gender and age, gender and “Other” career, and age and STEM career. In women, each additional year of age slightly *reduces* AQ score, while in men, each additional year of age slightly *increases* AQ. Career in a STEM area of work is associated in an increase in AQ scores in both genders, but the increase is greatest for men (for instance, according to the model, being a women (default gender) in a STEM career adds +1.15 points to the intercept score; men in STEM careers lead to a total of 1.15–0.44 points).

**Table 5:** The linear regression model relating covariates to AQ score. Female and Non-STEM used as reference levels. Correlation effect sizes = 0.1, 0.3, 0.5.

Model term		*B*	95% CI		p-value	Partial eta squared
	Intercept	20.35	20.23	20.47	<0.0001	0.19
Sex:	Male	-0.44	-0.67	-0.20	<0.0001	0.00
Age (Years)		-0.06	-0.06	-0.05	<0.0001	0.003
Occupation:	STEM	1.15	1.05	1.96	<0.0001	0.001
	Other	1.76	1.58	1.94	<0.0001	0.001
Interactions:	Male x Age	0.08	0.07	0.09	<0.0001	0.002
	Male x Other career	0.12	-0.21	0.45	0.46	0.00
	Male x STEM	-0.48	-1.04	0.09	0.10	0.00
	Age x Other career	0.00	-0.00	0.00	0.93	0.00
	Age x STEM	0.02	0.01	0.04	<0.0001	0.00
	Male x Age x Other	-0.02	-0.02	-0.01	<0.0001	0.00
	Male x Age x STEM	-0.01	-0.03	0.00	0.15	0.00

### Binary logistic regression

Binary logistic regression analysis was carried out to predict the probability of individuals falling above or below a cut-off value in AQ score. The cut-off used for this analysis was 32; in the original publication describing the AQ, 32 or greater was found in a general population sample to be the point at which sensitivity and specificity were optimized, with 80% of autism spectrum cases correctly identified and only 2% of controls qualifying as potentially undiagnosed cases [1]. This is in contrast to the other cut-off point reported in the literature of 26, which was derived from a study examining the use of the AQ as a screening tool at an adult autism clinic [3]; it was felt that the higher cut-off was more appropriate for a general population sample, rather than a sample seeking diagnosis. Thus, two categories were delineated: a high-risk group scoring above 32 that might be considered within the extended autism phenotype, and a low-risk category with low levels of autistic traits as self-reported on the AQ.

To determine which factors best differentiate individuals from the high- and low-risk groups, we again examined the available variables of Age, Sex, and Occupation. This model provides a significant improvement to the null fit, χ2(4) = 5068, p < 0.001 (Table 6).

**Table 6:** The binary logistic regression model relating covariates to AQ score. Female and Non-STEM used as reference levels.

Model term		*B*	Wald z-statistic	p-value	Odds Ratio
	Intercept	-2.43	-159.50	<0.0001	0.088
Sex:	Male	0.49	47.37	<0.0001	1.627
Age (Years)		-0.00	-5.75	<0.0001	0.998
Occupation:	STEM	0.44	30.76	<0.0001	1.558
	Other	0.40	37.29	<0.0001	1.494

Closer investigation of the model indicates that, while all terms are significant, Age, having an odds ratio of less than one, is the only term where an increase in the predictor leads to a decrease in the odds of the outcome (being in the high-risk category) increases. For Sex and Occupation, being male and being in a STEM career increase the odds of being in the high-risk category.

## Discussion

A dataset from the Channel 4 television website was used to examine the relationship between AQ and sex, occupation, age, and UK geographic region. Our predictions were strongly supported in that previously reported findings about different distributions of scores between males and females and between individuals working in STEM vs. non-STEM careers were replicated. In a sample of nearly half a million individuals, we found a moderate effect of sex on AQ, with males scoring higher than females by an average of 2.5 points. This replicates similar findings [2,16]. Further, with regard to occupation, we found that people working in STEM careers scored higher than people reporting non-STEM careers, again corroborating earlier findings [1,19,30]. In contrast, we found only a very small effect of age on AQ that is likely an artefact produced by the age-biased recruitment pool of the *Embarrassing Bodies* audience, summarized in Fig 1. We found no meaningful association of AQ with geographic location.

When sex, age, and occupation are entered into a multiple linear regression model, less than 4% of the variance in AQ in the current sample is explained, indicating that other unmeasured variables contribute to autistic traits as measured by the AQ. From previous work, we know that these unmeasured variables may include common genetic polymorphisms [31], prenatal testosterone [32] and brain structure [33].

The AQ was implemented on the Channel 4 website without the research team’s input, as initially this was not conceived as a research study, although the Ethics Committee retrospectively approved its use for research because the wording on the Channel 4 website alerted participants that the data would be used for research. The mean AQ score for the cleaned sample was 19.83 (SD = 8.71) is higher than that reported in the literature by approximately 3 points [2]. The dataset also has a positive skew. These findings reflect that there may be a bias in this self-selecting sample toward people who suspect they may be on the autism spectrum, as well as the likely inclusion of individuals with a clinical diagnosis of ASC in the sample. Participants were not asked as a part of the survey if they had ever received a diagnosis of ASC. Lack of this participant diagnostic information has made interpretation of novel results difficult, and has rendered it impossible for the binary logistic model described above to be assessed on predictive power of diagnosis, though we were able to use the cut-off values reported in the AQ literature. Variables such as whether participants have, or suspect they have, a diagnosis of ASC, or if they have a family member with a confirmed diagnosis, would be simple to collect in a future replication study, and useful for interpreting results. The Channel 4 team have agreed to include this information so that in a future replication of the current study, the effects of sex and STEM occupation can be studied in cases of ASC and controls separately. In future, research would also benefit from itemized response scores rather than AQ scores out of 50, so that subscale and item-level data could be analysed.

From this study we were able to replicate previous findings concerning the effect of sex and occupation on AQ scores, despite this not being a representative sample from the general population. This suggests these effects are robust and universal and allows us to conclude that traits commonly associated with autism are strongly linked to traits associated with being male and with STEM occupations, regardless of other factors. More generally, the use of a large data set in the current study demonstrates the value of collaborating with groups that have access to national and international survey platforms, and of collaboration between researchers and non-researchers.

Limitations to the current study include that the respondents were self-selecting rather than randomly selected. In addition, the only individuals who would have been aware of the invitation to volunteer to take the AQ were those who watched the late-night medical TV program *Embarrassing Bodies*, which appeals particularly to viewers who are not deterred by graphic details of medical syndromes, or who tuned in for this particular episode because it included an item on autism. The sample is relatively young compared to the UK population, and contains twice as many females as males. This is thus not a representative sample from the general population.

A further potential improvement in the design of the survey is in the selection options for demographic categories. The list of possible career options to select from was not compiled from national or international survey data, and nearly a quarter of participants selected the “Other” category. Given the age distribution of participants, it is possible that a large proportion of “Other” selectors were students, but this could not be confirmed. The “Other” category is likely a mixed group comprised of STEM and nonSTEM individuals. The same limitation applies to the location variable; locations were coarse-grained, and omitted large areas (e.g. London; East England). The presence of both an “Other” option and a “Prefer not to say” option caused a relatively large number of entries to be discarded during data cleaning. This means there was no opportunity to test the ‘Silicon Valley’ effect [21,34] in geographical areas that are enriched in jobs in Information Technology, which would be an interesting modification to include in a future replication study.

Another limitation to the current dataset is that there was no mechanism in the online platform by which to limit the number of times individuals could take the AQ, and no participant identifier in the data transferred to the research team that could be used to individuate results or remove potential duplicates. However, we assume that, in this case, the signal is greater than any noise. Evidence of an acceptable signal-to-noise ratio can be taken from the webmaster’s report, which states that the survey was taken 63,000 times during the hour-long show broadcast, suggesting that, at least during the first hour during which responses could be made, the majority of completed data entries are not duplicates.

Regarding statistical methodology, the ability of big data studies to identify subtle trends in a population can be misleading [15]. Historically, there has been criticism of null hypothesis significance testing, where it is argued that the method is an amalgamation of disparate techniques, that the results can be misinterpreted, that the test is employed in place of replication, and that the resultant conclusions could be unreliable [35–38]. Researchers concerned with the lack of reproducibility in the field of psychology have, among other things, criticized an overreliance on p-values, or the probability of rejecting the null hypothesis. In this study, we found that p-values, combined with large sample sizes, led to results that were difficult to interpret. For this reason, we also considered effect size, or practical significance, as a measure of the strength of an observed finding, as this measure is independent of sample size. Where appropriate, we also reported confidence intervals and odds ratios.

## Conclusions

In this study we confirm the effect of sex and STEM occupation on AQ, supporting earlier studies, and found no statistically meaningful effects of either age or geographical region on AQ. This study awaits replication in a big data study that controls for audience bias and diagnostic status. We conclude that autistic traits are consistently higher in males than females, and in those working in STEM than in non-STEM fields. These findings may have importance for understanding the male-biased sex ratio in autism [39,40] as well as the hyper-systemizing theory of autism [21,34,41].
